# Goals and Challenges of Stem Cell-Based Therapy for Corneal Blindness Due to Limbal Deficiency

**DOI:** 10.3390/pharmaceutics13091483

**Published:** 2021-09-16

**Authors:** Margarita Calonge, Teresa Nieto-Miguel, Ana de la Mata, Sara Galindo, José M. Herreras, Marina López-Paniagua

**Affiliations:** 1 IOBA (Institute of Applied Ophthalmobiology), University of Valladolid, 47011 Valladolid, Spain; tnietom@ioba.med.uva.es (T.N.-M.); adelamatas@ioba.med.uva.es (A.d.l.M.); sgalindor@ioba.med.uva.es (S.G.); herreras@ioba.med.uva.es (J.M.H.); 2 CIBER-BBN (Biomedical Research Networking Center in Bioengineering, Biomaterials and Nanomedicine), Carlos III National Institute of Health, 47002 Valladolid, Spain; 3 Castile and Leon Networking Center for Regenerative Medicine and Cell Therapy, 47008 Valladolid, Spain; 4 Ophthalmology Service, University Clinic Hospital, 47003 Valladolid, Spain

**Keywords:** blindness, cell therapy, CLET, cornea, limbal niche, limbal stem cell, LSCD, mesenchymal stem cell transplantation, MSCT, ocular surface

## Abstract

Corneal failure is a highly prevalent cause of blindness. One special cause of corneal failure occurs due to malfunction or destruction of the limbal stem cell niche, upon which the superficial cornea depends for homeostatic maintenance and wound healing. Failure of the limbal niche is referred to as limbal stem cell deficiency. As the corneal epithelial stem cell niche is easily accessible, limbal stem cell-based therapy and regenerative medicine applied to the ocular surface are among the most highly advanced forms of this novel approach to disease therapy. However, the challenges are still great, including the development of cell-based products and understanding how they work in the patient’s eye. Advances are being made at the molecular, cellular, and tissue levels to alter disease processes and to reduce or eliminate blindness. Efforts must be coordinated from the most basic research to the most clinically oriented projects so that cell-based therapies can become an integrated part of the therapeutic armamentarium to fight corneal blindness. We undoubtedly are progressing along the right path because cell-based therapy for eye diseases is one of the most successful examples of global regenerative medicine.

## 1. Introduction

Ophthalmology is among the first medical science branches that have benefited from stem cell-based therapy and regenerative medicine. Many facts can account for this success, such as easy accessibility to the stem cell niches (especially those located in the anterior segment of the eye); a relatively easy follow-up of the applied therapies; the fact that the eyes are paired, non-vital organs; and the immune-privileged nature of the intraocular tissues and cornea [1].

Of all of the potential stem cell niches in the visual system [1], those located at the ocular surface are the most accessible for study and extraction of the stem cells, and they can be repaired if they fail and produce disease. Consequently, second only to hematopoietic stem cell transplantation, regenerative medicine of the ocular surface is the most well developed. In fact, epithelial stem cell transplantation to repair the corneal surface is the most widely used stem cell-based therapy in clinical medicine.

The anterior segment of the eye is composed of the ocular surface, the anterior sclera, the corneal stroma and endothelium, the anterior and posterior chambers containing the aqueous humor, the anterior uvea composed of the iris and ciliary body, and the crystalline lens. The posterior segment of the eye consists of the posterior uvea containing the choroid, the retina, the optic nerve head, the vitreous body, and the posterior sclera. The concept of the ocular surface includes those structures and tissues directly exposed to the environment. Thus, the ocular surface is comprised of the overlying tear film, superficial cornea (the epithelium; Bowman’s layer; and for some eyes, the superficial stroma), conjunctiva, and the corneoscleral limbus. The main tissue at the ocular surface is the epithelium because, in general, only epithelial tissues have direct contact with the environment through either the skin or diverse mucosal sites, including the ocular surface.

Several potential stem cell niches are present in the ocular surface. Some are still under investigation, and in the future, these sites may enable the isolation of human adult stem cells from the conjunctiva, corneal stroma, and/or the meibomian glands [2]. The procedures for isolating the stem cells and the knowledge learned from them could provide fundamental insights and new regenerative therapies for many diseases, such as cicatricial conjunctivitis (i.e., Stevens–Johnson syndrome and the associated spectrum), conjunctival trauma, and meibomian gland disease, for which there are currently no effective, long-term treatments.

In the ocular surface, the most well-known and documented stem cell niche is located at the limbus, where the transparent cornea transitions to the opaque sclera and is overlain by the conjunctiva. Stem cells in that location have been identified, extracted, and used therapeutically in attempts to repair the severe corneal pathology when these cells and/or their niche are damaged. The goals and challenges that this therapy constitute are the topic of this review.

## 2. The Past: The Beginnings of Stem Cell-Based Therapy for Corneal Failure

**Figure 1:**
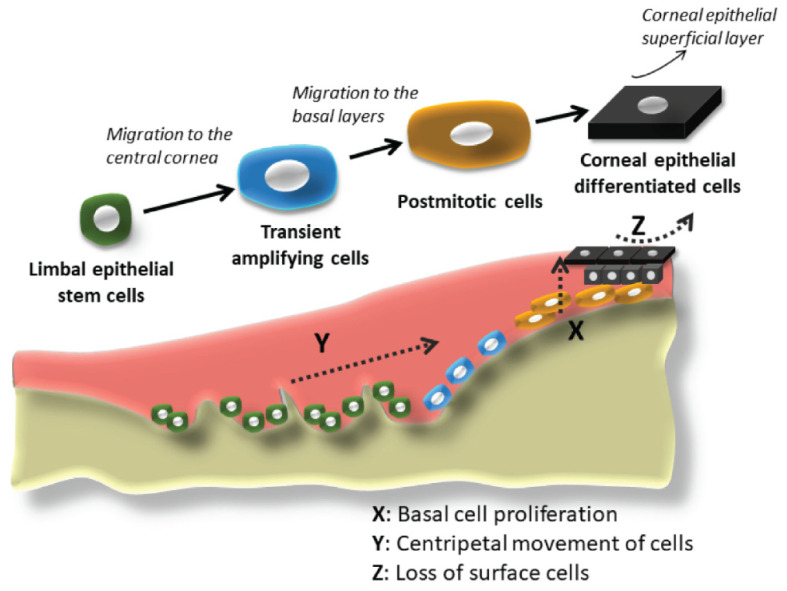
Graphical representation of the limbal stem cell niche with all of the cell states implicated in the corneal epithelium regeneration (limbal epithelial stem cells, transient amplifying cells, postmitotic cells, and corneal epithelial differentiated cells). The X, Y, Z hypothesis published by Thoft and Friend in 1983 [8] is also represented, presenting the three different phenomena that allow the corneal epithelial cell mass to remain constant. X: proliferation of basal epithelial cells; Y: contribution to the cell mass by centripetal movement of peripheral cells; Z: epithelial cell loss or constant desquamation from the surface.

Much of the knowledge regarding the location of corneal epithelial stem cells in the limbal area came from clinical observations and subsequent experimentation in the field of corneal epithelial wound healing, coupled with the research in the field of skin stem cells. At the beginning of the 20th century, ophthalmologists and scientists began studying the first phenomenon in the process of corneal epithelium wound healing: sliding or migration of the surrounding epithelial cells (reviewed in Schwab, 1999 [3]), and the subsequent cell replication and proliferation to generate replacement cells. In 1944, Ida Mann demonstrated pigment movement from the limbus in a rabbit model of corneal injury [4], which matched the observations by clinicians that epithelial lines seemed to move from the peripheral cornea to the wound site in the central cornea. Friedenwald and Buschke, also in 1944, demonstrated that the mitotic index of corneal epithelium tended to be higher towards the periphery of the cornea [5]. Maumenee, in 1964, was the first to suggest that the corneal epithelium could be regenerated efficiently from the limbal epithelium and, to a lesser extent, from the conjunctival epithelium [6]. In 1971, Davanger and Evenson described the rapid movement of peripheral cells in response to an acute central defect [7]. They referred to the source of the migrating cells as the “pericorneal papillary structure”, which today, is called the palisade of Vogt, located at the limbus. They proposed that the essential role of the migrating cells was the renewal of the corneal epithelium and suspected that, to maintain corneal transparency, the source of these cells had to be in the nearest vascular stroma, i.e., the limbus. In 1983, Richard Thoft and Julie Friend published their famous X, Y, Z hypothesis [8], where they established that there are three different phenomena that kept the corneal epithelial cell mass more or less constant under physiological conditions: The X component of this hypothesis is the proliferation of basal epithelial cells and migration toward the surface. The Y component is the contribution to the corneal epithelial cell mass by the centripetal movement of peripheral cells. The Z component is the loss of epithelial cells through normal desquamation. When the hypothesis was proposed, the Y component was not proven, though there was evidence of it. For instance, following corneal transplantation, the centripetal movement of cells from the host peripheral cornea replaced the loss of the corneal epithelial cells from the donor transplant [9]. This elegant hypothesis was validated later as the Y component was proven to be the mass of limbal stem cells. The simplicity of Thoft’s statement that “corneal epithelial maintenance, essential to avoid pathology, could be defined by the equation ‘X + Y = Z’” could not be truer to this day [8] (Figure 1).

In 1986, Schermer et al. offered strong evidence that the location of the corneal epithelial stem cells was at the limbus [10]. By studying keratin expression patterns, they demonstrated that limbal basal cells were less differentiated than corneal epithelial basal cells. This supported the clinical observation of the centripetal migration of corneal epithelial cells. They concluded that corneal epithelial stem cells were likely located in the limbus and, furthermore, that the basal corneal epithelial cells corresponded to cells then known as “transient amplifying cells” because the more apically located epithelial cells were terminally differentiated [10].

Based on those early studies, multiple researchers have contributed to better defining the limbal epithelial stem cells along with other cells located in the limbal niche and the niche itself. Additionally, a wide range of markers have been identified to define each of the different cell types in that niche [11].

**Figure 2:**
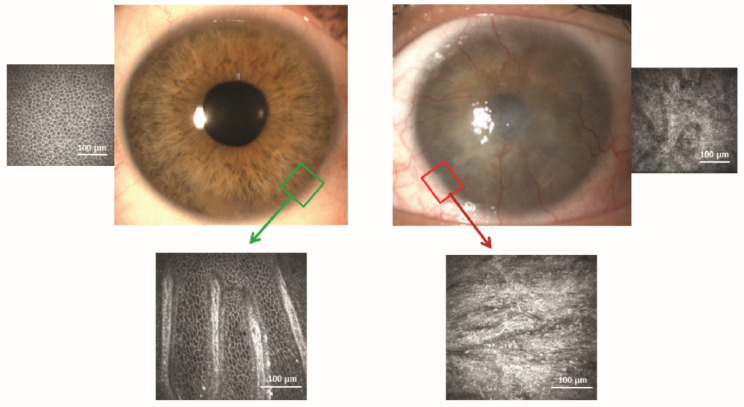
(**Top Left**) Location of the limbal niche (**green box**) in the normal eye of a 26-year-old man. (**Lower Left**) Confocal image of the limbal niche that is responsible for maintaining the corneal epithelial phenotype. (**Top Right**) The left eye of the man suffered a chemical injury 2 years before. The injury destroyed his limbal niche (**red box**). Consequently, the corneal epithelium has a conjunctival phenotype derived from the adjacent conjunctiva. (**Lower Right**) Confocal image of the damaged limbal niche.

Clinically, ophthalmologists have now defined the term “limbal stem cell deficiency” (LSCD) to describe what had previously been called “conjunctivalization” or “neovascular pannus” before the presence of stem cells was even suspected. LSCD describes certain severe conditions in which cells of the damaged ocular surface epithelium are not replaced with centripetally migrating cells derived from the limbal niche. Consequently, the conjunctiva encroaches upon the cornea, replacing the injured corneal epithelium and preventing further irreversible structural damage such as infection, stromal necrosis, or even perforation. While conjunctival overgrowth results in the loss of functionality, i.e., visual loss due to corneal opacification, it prevents the loss of corneal anatomical integrity that could ensue otherwise (Figure 2).

Primary LSCD is currently defined as an end-stage pathology resulting from multiple diseases that destroy the corneal niche located at the corneoscleral limbus. Secondary LSCD refers to the loss of the resident corneal epithelium stem cells even though the limbal niche remains intact. LSCD can be hereditary, e.g., congenital aniridia, or acquired, e.g., immune-mediated diseases such as Stevens–Johnson/toxic epidermal necrolysis, atopic keratoconjunctivitis, rosacea-related pathology, and non-immune mediated pathologies such as chemical injuries [12].

LSCD results in recurrent corneal epithelial ulceration, conjunctivalization (pannus with superficial neovascularization), and opacification because of the inability of the limbal niche to renew the corneal epithelium. It is well established that LSCD, when extended and/or severe, usually leads to corneal opacity and subsequent blindness with accompanying symptomatology. Diseases leading to LSCD and LSCD itself are extremely difficult to manage, and in most instances, they need proper, aggressive medical therapy before proceeding with surgical treatment [12].

Understanding the functionality of the limbal niche and the consequences of its pathology explain why corneal transplants in patients with ocular surfaces diseases in which LSCD is concomitantly present are doomed to fail. Failure of the initial transplant and of subsequent ones is due to the incapacity of the host limbus with LSCD to replace the epithelium of the transplanted corneas as the cells are lost through desquamation during the post-transplant period. At present, ophthalmologists have now agreed that corneal transplantation is not a viable primary solution if extensive LSCD is present because transplantation does not replace the damaged corneal epithelial stem cells [13]. Repair of the limbal stem cell niche must be achieved first. Consequently, ophthalmologists began to design strategies to remove limbal tissue from the fellow healthy or the less affected eye of the patient or, alternatively, from allogenic sources such as cadaveric eyes or healthy living relatives, to transplant into the LSCD-affected eye.

Kenyon and Tseng pioneered the transplantation of limbal tissue in 1989. They reported good results at 6 months after surgery for 21 of 26 LSCD cases subjected to limbal autograft transplantation in which the limbal tissue was removed from the less injured eye [14]. Following that report, large auto- and allo-limbal grafts were performed for LSCD, but soon, two problems became evident: the risk of limbal failure in the donor eye in case of autografts, and the need for potent and protracted immunosuppression for allografts.

Since the initial years of limbal tissue transplantation, the methodology has evolved and is now performed as keratolimbal autografts, keratolimbal allografts, conjunctival limbal autografts, or conjunctival limbal allografts. Allografts must be chosen for cases of bilateral disease, and the donor source is from a cadaver or from a living related or non-related donor. For all of the transplantation methods, keratoplasty can be performed either concomitantly or following a delay, though in both cases the outcomes are highly variable, ranging from very poor to good.

One important problem with transplantation of allogeneic limbal tissue is the need for long-term systemic immunosuppression, even in HLA-matched donors, without which long-term survival of the transplanted niche tissue is unlikely [15]. Furthermore, two limbal grafts measuring approximately 6 mm at the limbus (3 h clock quadrants) and extending 5–8 mm posterior to the limbus are removed from the healthy donor eye. This extensive extirpation could lead to pathology in the donor eye [16]. In any case, these procedures are still in current clinical practice [17].

Tissue-based transplantations require a significant amount of tissue that is derived from the fellow eye for transplantation into the damaged eye. An alternative approach is to harvest small amounts of healthy limbal niche tissue from the donor eye and then to generate more autologous stem cells through tissue culture (see below). However, this approach requires the use of good manufacturing practice (GMP) cell culture facilities, which are expensive to maintain and may not be generally available to all ophthalmologists and patients who need them. Based on these limitations, in 2012, Virender Sangwan and his group in India pioneered another tissue-based technique of tissue transplantation known as simple limbal epithelial transplantation (SLET) [18]. It uses a smaller limbal tissue biopsy (3–4 mm) from the contralateral healthy eye (autologous). The tissue is then cut into 8–10 tiny pieces and placed on top of an amniotic membrane that was previously glued by fibrin to the scraped, diseased corneal bed of the recipient eye. The more than 30 reports published so far suggest good mid-term results as long as autologous tissue is used [19]. A modification of this technique uses a double layer of cryopreserved amniotic membrane to sandwich the limbal cells [20] and has been approved by the US Food and Drug Administration (FDA). As an alternative for bilateral cases in which healthy autograft donor tissue is not available, the alloSLET, using allogeneic tissue, was recently introduced by Shanbhag et al. with apparent good results [21]. It is important to understand that no matter how small the allogeneic tissue is, systemic immunosuppression is still needed. With alloSLET, Shanbhag et al. used a pulsed intravenous immunosuppression regimen for long term, i.e., more than 2 years, or the usual oral protocols used for cultivated limbal cells (see below) for an amount of time not yet specified [21].

To reduce the high rate of immune rejection of tissue-based transplants, Pellegrini et al. described for the first time, in 1997, stem cell-based therapies (as distinct from tissue-based therapies) that transplanted cultivated, autologous limbal epithelial cells that were extracted from small biopsies of the contralateral donor eye [22]. Although it was described for just two patients and did not follow GMP techniques, this cultured cell approach represented a significant breakthrough in regenerative medicine.

## 3. The Present: Available Stem Cell-Based Therapies

At the beginning of the 21st century, bilateral cases of LSCD continued to be a challenge because allogenic limbal tissue transplantation was generally unsuccessful without long-term immunosuppression. Furthermore, the availability of stem cell-based transplantation was becoming more limited due to the implementation of new regulatory guidelines that guaranteed safety but restricted the conditions under which auto- and allograft treatments could be used. This section describes the stem cell-based therapies currently available for both unilateral and bilateral LSCD.

### 3.1. Cultivated Limbal Epithelial Transplantation

Limbal epithelial stem cells, the adult stem cells of the corneal epithelium, have been widely used in both preclinical and clinical studies for LSCD therapy, as described in this review. The transplantation of limbal epithelial cells facilitates ocular surface regeneration, and consequently, it improves the prognosis of a subsequent corneal graft [23,24,25,26,27,28,29,30,31]. Molecular and functional characterization of these cells and their use for LSCD therapy are also being vigorously investigated in preclinical and clinical ophthalmological research.

Although the fate of transplanted limbal stem cells on the ocular surface is still not certain, some potential mechanisms of action have been identified: (1) donor cell migration to the host niche and subsequent regeneration of the niche before corneal epithelial repair, (2) donor cell creation of a new pseudo-niche before regeneration of the corneal epithelium, (3) donor-cell stimulated regeneration of the corneal epithelium through paracrine or direct interaction, and (4) donor transit-amplifying cells acting as the principal cells responsible for regeneration of the host epithelium [32,33,34].

After the first publication by Pellegrini et al. in 1997 of two patients treated with cultivated limbal cells extracted from a small (approximately 2 × 2 mm) biopsy of limbal tissue from the contralateral healthy eye [22], other authors began developing similar techniques and published different reports on autologous cultivated limbal epithelial transplantation (CLET). In 2008, the European Medicines Agency (EMA) granted orphan drug status for a product that provided “ex vivo expanded autologous human corneal epithelium containing stem cells for the treatment of corneal lesions, with associated corneal (limbal) stem cell deficiency, due to ocular burns” [35]. In 2010, Rama et al. reported a 76.6% success rate in the 2–3 years of follow-up of 112 cases treated with a stem cell-derived product that was similar to the one used in 1997 by the same group [36]. In 2015, that product received the trade name of Holoclar and it became the first stem cell-based therapy to be approved by the EMA with a conditional marketing authorization pending on further clinical evidence of safety and efficacy [37,38]. Holoclar has not yet achieved approval for standard commercialization; however, if and when it is, it will only be authorized for treatment of chemical injuries with stem cells derived from an autologous source.

The great advancement of Pellegrini and Rama’s group [22,36,37] prompted many other groups to start developing similar protocols with some modifications in culture, evaluation procedures, and surgical techniques. There are consequently some published reports, especially in Asia and Europe [39], on autologous CLET with success rates varying from 60% to 100% [40,41].

Soon after starting treatments with autologous CLET, ophthalmologists realized that there were many cases of bilateral LSCD where this therapy was not an option, usually because the disease or trauma caused bilateral LSCD, leaving no healthy limbal tissue available for biopsy. Then, encouraged by the good results of autologous transplants, ophthalmologists began investigating allogeneic CLET. The first attempts of allogeneic CLET in patients with LSCD were by Schwab in 1999 [3], who published two partially successful allogeneic cases that utilized stem cells derived from living relatives of the patients, and by Koizumi et al. (Kinoshita’s group) in 2001 [42]. The latter publication was a retrospective case series, reporting success in 10 of 13 eyes followed for 11.2 months. Cells were obtained from cadaveric limbal explants, cultured on a carrier composed of denuded amniotic membrane coated with a layer of 3T3 fibroblasts to assist epithelial cell growth. The cultivated epithelium consisted of four to five stratified cell layers and was positive for corneal specific keratins (K3/12). None of the patients were immunosuppressed, but only three eyes suffered epithelial rejection.

In the following years, other authors began using allogeneic limbal tissue from cadaveric sources. These were used mainly for cultivation and extraction of limbal epithelial cells. Initially this was conducted in regular tissue culture laboratories; however, stem cell-based therapies are considered to be medicinal products. Therefore, it was necessary to move production into GMP-based cell culture facilities to comply with the same regulatory principles as conventional drugs [43,44].

The techniques for the cultivation of allogeneic stem cells and surgeries are diverse but are the same as those for autologous cells. The main difference is that the patients receiving allogeneic cell-based transplants need to be systemically immunosuppressed but require only one drug and endure a shorter period of immunosuppression (12 months maximum) [40] than required for high risk corneal transplants or for allogeneic limbal tissue transplantation (full segments or SLET). The best culture, surgical, medical, and evaluation protocols, however, have not yet been defined for either autologous or allogeneic CLET.

In 2015, Zhao et al. published a systematic review and meta-analysis of ex vivo CLET using amniotic membranes as a substratum in LSCD [45]. In 18 publications involving 572 eyes (562 patients), the success rate was about 67% for both autografts and allografts, provided that the allografts receive systemic immunosuppression, which were less intense than required for the transplantation of non-cultured allografts [45]. These results were consistent with previous reviews by Baylis et al. [28] and Shortt et al. [46] of 28 reports and 17 reports, respectively, that compared autografts and allografts. Another more recent meta-analysis by Mishan et al. of autologous versus allogeneic CLET included 30 studies, with sample sizes ranging from 6 to 200 and follow-up periods of 0.6–156 months [41]. Of the 1306 eyes, 982 (75.2%) received autografts and 324 (24.8%) received allografts from living or deceased donors. The meta-analysis revealed that the odds of success were similar for both CLET procedures.

One prospective comparative study stated that allogeneic CLET yields far worse results than autologous CLET [47]. However, the authors of this study failed to give the necessary immunosuppressive treatment to their allogeneic transplantation patients, and this most likely explains why these patients had unsuccessful transplantations.

Another recent meta-analysis of 40 studies (2202 eyes) concluded that autologous grafts had a higher rate of ocular surface restoration and a lower rate of complications than allogeneic grafts [40]. In their analysis, the authors combined the outcomes for cases treated with allo-CLET and allo-limbal tissue transplantation. However, these two approaches are fundamentally independent and incompatible with one another for the purpose of being combined in a meta-analysis. Thus, their finding of differences in the rate of ocular surface restoration and in the rate of complications for autologous and allogeneic treatments was flawed.

**Figure 3:**
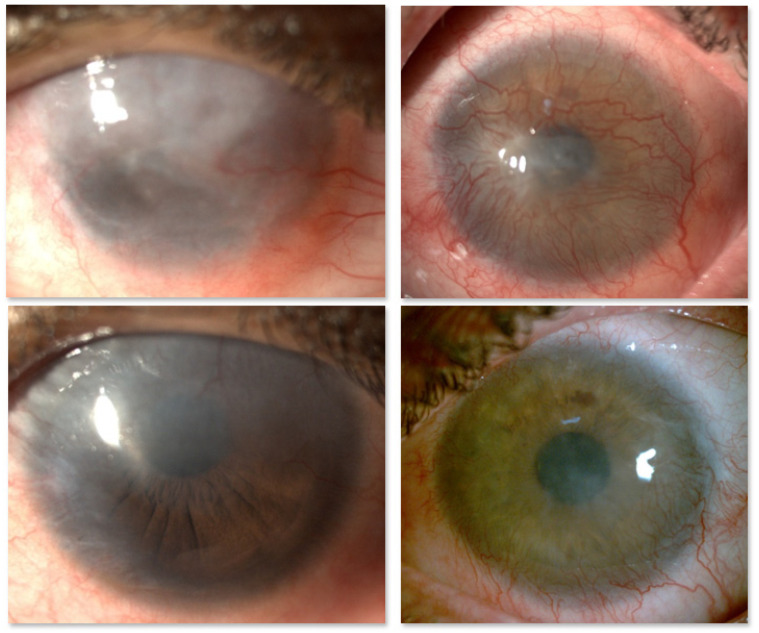
Chemical injury in two different patients, before (**upper panel**) and 12 months after (**lower panel**) successful cultivated limbal epithelial transplantation (CLET). The source of the limbal epithelial cultivated stem cells was autologous, from the fellow healthy eye (**left panel**) or allogeneic from cadaveric limbal ring (**right panel**).

In conclusion, CLET results seem to be similar whether the cell source is autologous or allogeneic (Figure 3). Even though the objective assessment of outcome is difficult because the cases are extremely variable in every possible aspect, the success rates range between 60% and 100%. Additionally, following LSCD treatment, eyes with significant stromal and/or endothelial damage require subsequent corneal transplantation. In these cases, visual acuity is not a good indicator of success [24], something that it is very difficult for patients, relatives, and even referring clinicians to understand.

However, without a doubt, significant clinical improvements have been achieved in the treatment of ocular surface pathology due to LSCD. These improvements are based on the evolution of our understanding of the disease origin and advances in treatment methodology, all of which have improved the prognosis, with or without the need for subsequent corneal transplants. Consequently, patient quality of life has vastly improved [34].

In terms of safety, xenobiotic-free conditions are now broadly used to minimize the risk of diseases or immune reaction [45,48]. For instance, in the case of allogeneic grafts, CLET reduces the exposure of the host to non-self-antigens. With the in vitro expansion of cells from a small biopsy, most antigen-presenting Langerhans cells and other cells found in the normal limbal stem cell niche are lost [49]. Importantly, tumorigenic events have not been reported in either preclinical studies or in clinical practice.

### 3.2. Autologous Non-Limbal Epithelial Cell Transplantation

Another potential solution for bilateral cases of LSCD, where autologous limbal tissue is not available, is the transplantation of cultured cells from autologous non-limbal tissues. Different preclinical studies have demonstrated that cultivated oral mucosal epithelial cell transplantation (COMET) in the treatment of the ocular surface in experimental models of LSCD in rabbits reduces corneal epithelial defects, corneal opacity, and vascularization [50,51,52,53]. However, some corneas had irregular epithelial surfaces associated with peripheral neovascularization after COMET [50,51]. In 2004, Nakamura et al. published the first clinical studies that used autologous COMET [54]. In a recent review of 24 publications between 2004 and 2019 [55], the authors concluded that COMET is the most frequently used non-limbal autologous cell procedure in the treatment of bilateral LSCD, possibly because it eliminates the risk of graft rejection and thus avoids the need for immunosuppression. The COMET approach has been preferentially performed in Japan, and based on published cases from the last 15 years, they offer promising mid-term results with a stable ocular surface reported in 70.8% of LSCD eyes [55]. However, neo-angiogenesis following transplantation is a drawback associated with this procedure, but solutions for this problem have been proposed. For example, the mucosal epithelial cells can be co-cultured with limbal mesenchymal niche cells instead of the usual 3T3 cells [56]. The oral mucosal cells obtained from this alternative culture system seem to be less likely to induce postsurgical neovascularization and therefore improve postsurgical outcomes.

Although the molecular mechanisms of COMET are still unknown, the transplanted cells remain in the cornea for at least 24 weeks in rabbits [51] and up to 22 months in humans [57]. In addition, the expression of corneal epithelial marker K3 [52,57] and the limbal epithelial marker p63 [51,57] were found in rabbit and human corneas after COMET. In contrast, the corneal epithelial marker K12 showed variable expression, being absent from many corneas but present in others after COMET [51,57].

It is extremely difficult to make comparisons among techniques. Nevertheless, Wang et al. compared the success rate of COMET to allogeneic CLET [58]. The success rate of COMET, 52.9% (18 of 34 eyes), was lower than that for allogeneic CLET, 71.4% (30 of 42 eyes). The difference was attributed mainly to a higher incidence of postoperative complications with COMET due mostly to persistent epithelial defects. The authors attributed the different results to the unique morphology, function, and microenvironment of oral mucosal epithelium compared to the limbal epithelium despite the expression of similar gene markers.

Another more recent review by Samoila et al. in 2020 [59] compared the clinical outcomes of allogeneic CLET (18 publications between 2005 and 2019) and COMET (11 publications between 2004 and 2019) for total bilateral LSCD. They identified the advantages of COMET, including the lack of graft rejection (as it is autologous), the achievement of cell culture in a shorter period of time, and the absence of keratinization over a prolonged time span. In addition, tumorigenic events have not been reported. However, they also reiterated the previously identified shortcomings such as the lower success rate of the oral mucosal epithelial cells due to the higher incidence of post-operative persistent epithelial defects and subsequent graft failure, and lower cell proliferation and differentiation activities. They concluded that allogenic limbal epithelial stem cells may have a better ability to form a stable and integrated corneal epithelium.

In summary, there is no current agreement regarding the preferred use of COMET or allogeneic CLET for total LSCD patients. Most publications agree that allogeneic CLET should be prioritized over COMET because the limbal epithelial cells may have a better capacity to maintain ocular surface stability, provided that immunosuppression is used, which is not necessary with COMET.

### 3.3. Allogeneic Non-Limbal Stem Cell-Based Transplantation

The only non-limbal allogenic stem cells that have been used clinically are mesenchymal stem cells (MSCs). As recently documented, MSCs were the most commonly used stem cell type for cellular and tissue-engineering therapies in Europe between 2016 and 2017 [39]. This is mainly due to the capacity of MSCs to produce growth factors, to modulate immune and inflammatory properties, and to differentiate into multiple cell lineages depending on the environmental signals [60]. MSCs secrete epithelial growth factor, which increases corneal epithelial cell proliferation [61,62]. Other growth factors secreted by MSCs include hepatocyte growth factor, fibroblast growth factor, and nerve growth factor [63,64]. To modulate the inflammatory response, MSCs secrete anti-inflammatory molecules, such as transforming growth factor β, thrombospondin 1, and tumor necrosis factor-stimulated gene-6 [61,65]. Other secreted molecules have either pro- or anti-inflammatory effects depending on the microenvironment. These include interleukin 6, which is increased in the presence of damaged corneal epithelial cells [63]. In addition, MSCs can produce antioxidant enzymes such as dioxygenase-2,3-indolamine and cyclooxygenase 2 in the oxidative stress environment associated with damaged corneas [66]. MSCs can also reduce T lymphocyte proliferation and can inhibit differentiation of immature macrophages to mature (active) macrophages [67,68,69], thus regulating the immune response by reducing rejection and inflammatory reactions [70].

When applied to the ocular surface in preclinical animal models of LSCD, MSCs exerted potent reductions of inflammation, corneal opacity, and neovascularization, all while promoting re-epithelialization [66,71,72,73,74,75,76,77,78]. Other important preclinical studies showed that MSCs can migrate specifically to the damaged corneolimbal tissues [73,78,79,80] and can improve the therapeutic response of the ocular surface affected by LSCD.

Regarding safety-related issues in preclinical studies, the transplantation of MSCs to treat LSCD does not induce adverse events, and no toxicologic effects have been reported [73,74,75,76,78,81]. Nevertheless, several preclinical works to analyze the tumorigenic potential of MSCs have been reported, confirming that the transplantation of this type of cell does not induce tumorigenesis in either healthy vital organs or in damaged target organs [82,83,84,85,86].

MSCs also have potential advantages over limbal epithelial cells because they can be easily obtained from many tissue types without the dependence of deceased donors. Additionally, they can be cultured in vitro, achieving clinical scales in a short period of time by less expensive procedures than those required for limbal epithelial stem cells. Importantly, 100% of the MSCs in a transplant are stem cells, while only a variable proportion of the cells cultured for CLET are indeed stem cells, as they are extracted from limbal tissue, and the limbal epithelium consists of only about 5–10% of stem cells [87]. Finally, the MSCs can be cryopreserved without loss of potency and allogeneic MSCs can be transplanted without the need of host immunosuppression [88].

**Figure 4:**
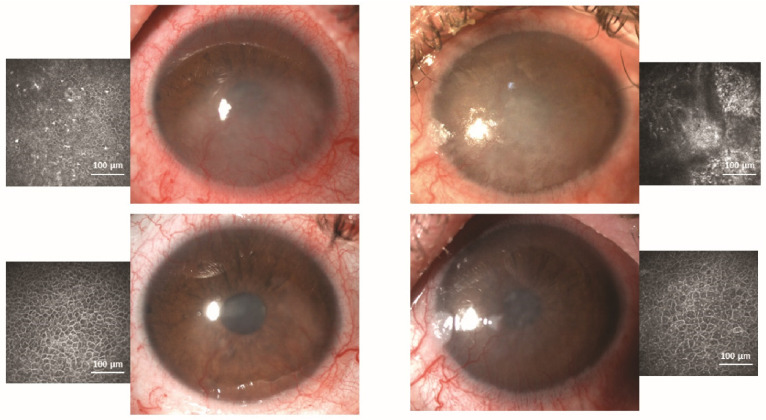
Bilateral limbal stem cell deficiency due to Stevens–Johnson syndrome. The right eye (**left panel**) was randomized to receive allogeneic limbal stem cell transplantation (CLET), and the left eye (**right panel**) received a mesenchymal stem cell transplantation (MSCT). The upper panels show that both corneas have a mixed epithelial phenotype as imaged by in vivo confocal microscopy. The lower panels show the same eyes after 12 months. Both corneas have an epithelial phenotype in the central cornea. Both transplants were successful.

The first and only published clinical use of MSCs for LSCD was by Calonge et al. in 2019 [89]. These authors designed a 12-month proof-of-concept double-masked clinical trial in which 22 patients with severe and total LSCD were randomized to either allogeneic bone marrow-derived mesenchymal stem cell transplantation (MSCT) or allogeneic CLET. All patients had immunosuppression with one drug for one year to maintain investigator masking, even though MSCT did not require it. Both cell types produced similar results and were equally safe. MSCT was successful in 85.7%, and CLET was successful in 77.8%. The central corneal epithelial phenotype evaluated by in vivo confocal microscopy improved in 71.4% and 66.7% of MSCT and CLET cases, respectively (Figure 4). There were no adverse events related to MSCT or CLET [89]. Therefore, the tumorigenic potential of MSCs has been discounted. If these good results are corroborated in a large series of patients, MSC therapy is deemed a safe, efficacious alternative for both unilateral and bilateral LSCD cases.

Finally, Table 1 summarizes the distinctive characteristics and success rates of different published stem cell-based therapies that report comparative studies among different sources of cells.

### 3.4. Regulatory Status of Stem Cell-Based Therapy for Treatment of LSCD in Different Countries

Autologous and allogeneic CLET are now performed in several European centers. The procedures require permission from national or European regulatory agencies and must be conducted within GMP guidelines. In the European Union (EU), the specific legal framework for advanced therapy medicinal products (ATMPs) was established by the European Commission in Regulation EC No. 1394/2007 and is regulated by EMA [43,101]. Currently, only Holoclar, the first and only stem cell-based therapy for autologous LSCD, has a conditional marketing authorization with orphan designation in the EU. Moreover, although the UK has recently left the EU, the UK Medicines and Healthcare products Regulatory Agency (MHRA) continues regulating ATMPs following the European regulations. In this regard, Holoclar is still authorized in the UK. In other European countries outside the EU, the regulation of stem cell therapy is at the national level. In Turkey, where autologous CLET has been conducted, the stem cell therapy must follow the guidelines entitled “The Guide to Non-embryonic Cell Studies for Clinical Purposes”, prepared by The Scientific Advisory Board of Stem Cell Transplantations [102]. In the US, stem cell-based therapies are regulated by the FDA and, similar to the EU, cell-based products are considered as biological drugs when they are subjected to more than minimal manipulation or non-homologous use. Currently, in the US, there are no FDA-authorized cellular therapy products for ophthalmic indications [20,103]. Nevertheless, the SLET, not considered as a cell-based therapy, was approved by the FDA for clinical use in 2014 [20]. In addition, autologous or allogeneic limbal epithelial stem cells expanded ex vivo on human amniotic membrane were designated in 2005 as orphan drugs by the FDA to treat LSCD. Allogeneic ABCB5-positive limbal epithelial stem cells were similarly designated in 2019. In Japan, stem cell-based therapy products are regulated by the Pharmaceuticals and Medical Devices Agency (PMDA), Ministry of Health, Labour, and Welfare. These medicines are classified as regenerative medicinal products [104,105]. Autologous CLET, allogeneic CLET, and autologous COMET clinical trials have been developed under Japanese legislation. In 2020, commercial use of a stem cell-based regenerative medicinal product, Nepic, was authorized by the PMDA to treat LSCD, and it is currently manufactured by the Japan Tissue Engineering Co., Ltd. (Gamagori, Japan). In India, the National Stem Cell Guidelines regulate stem cell therapies. They were jointly written in 2007 by the Indian Council of Medical Research and the Indian Department of Biotechnology. The guidelines were revised in 2013 and 2017, and since then, stem-cell-based products derived from substantial, or more than minimal, manipulation have been considered as drugs. However, these amendments exclude minimally manipulated stem cells from the category of drugs [106,107]. At present, in India, there are no approved indications for stem cell-based therapy apart from hematopoietic stem cell transplantation. Therefore, any other stem cell-based therapy must be treated as investigational and conducted only in the form of a clinical trial after obtaining regulatory authorization. Finally, in Australia, cell-derived products are regulated under the Therapeutic Goods Administration regulatory framework for biologicals and must comply with the Therapeutic Goods Order No. 88 [108] and the Australian Code of Good Manufacturing Practice [109].

## 4. The Future: Challenges to Overcome in Stem Cell-Based Therapies

The ideal goal in the management of corneal blindness due to LSCD is to restore the architecture of the limbal niche so that new stem cells, coming from internal and/or external sources, can repopulate the niche and can replicate in a successful way such that the corneal epithelium can be regenerated with its original properties: transparency, uniformity, and self-renewing capacity.

**Figure 5:**
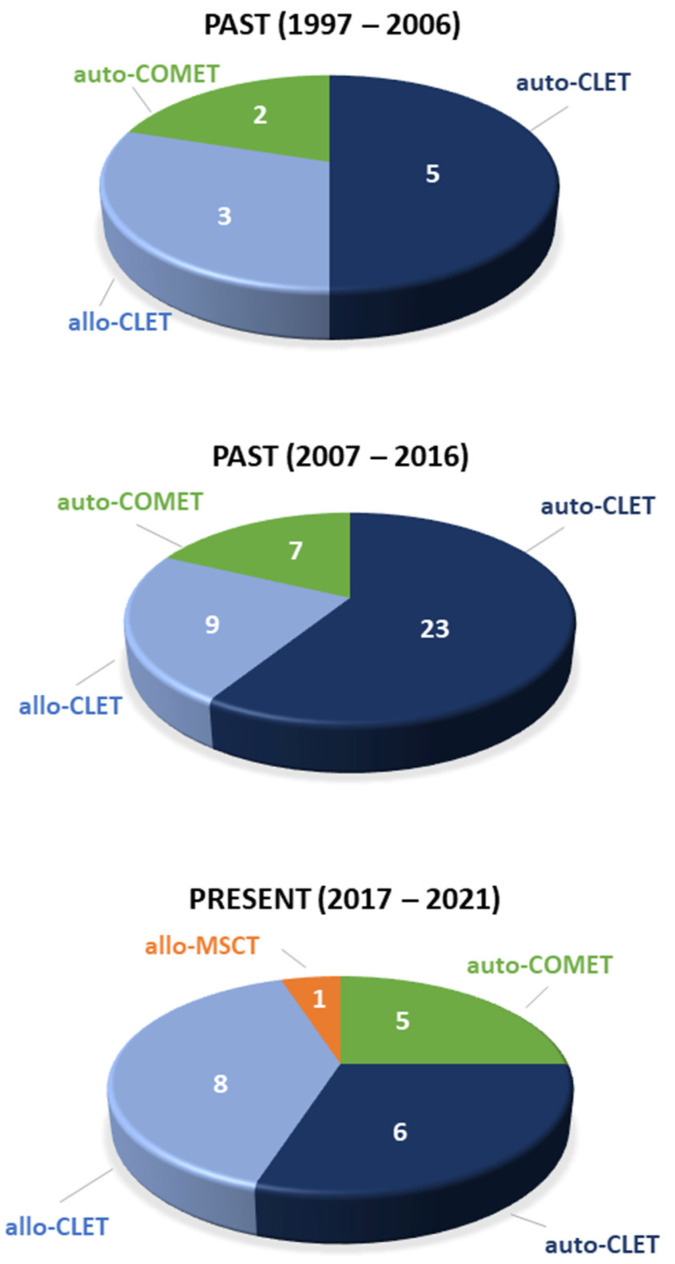
Evolution of stem cell-based therapies to treat corneal failure due to limbal stem cell deficiency from 1997 until the present. The data are for only published clinical trials. CLET, cultivated limbal epithelial transplantation; COMET, cultivated oral mucosal epithelial cell transplantation; MSCT, mesenchymal stem cell transplantation; auto, autologous; allo, allogeneic.

We have made enormous progress since our preceding colleagues began tissue-based techniques and then stem cell-based techniques (Figure 5). The farthest we have achieved at present is to transplant stem cell-containing tissues or cultivated stem cells extracted from those tissues.

Selection of the best techniques by comparisons among the different therapeutic approaches available at present can hardly be made. Variations in LSCD diagnosis and grading, cell culture protocols, transplantation techniques, postoperative management, evaluation of success vs. failure, etc. are so great that comparative analyses would be inaccurate and unfair. Additionally, there are still no clear answers as to how these therapies work when the stem cells or tissues are transplanted into the human eye. Thus, we have a long way to go as to be able to anatomically and functionally repair a destroyed limbal niche and the stem cells that normally reside there. With no intention of being comprehensive, we selected and described some examples of the relevant clinical and pre-clinical challenges that must be faced and overcome below.

### 4.1. Clinical Challenges

At present, autologous and allogeneic CLET are performed in several specialized centers around the world, and COMET techniques are being redesigned to offer better results. MSC-based therapies are showing promising results while waiting for larger confirmatory clinical trials [89].

Clinicians have not yet reached full agreement about the clinical stages and gradations of LSCD or even about how to diagnose LSCD [12,110], although efforts are being made [12]. For example, some defend that clinical slit-lamp biomicroscopy findings as observed by experienced ophthalmologists, e.g., loss of limbal normal features, whorl-like epitheliopathy, superficial opacification, neovascularization, persistent epithelial defects, and fluorescein late staining, are sufficient evidence to support a diagnosis of LSCD; however, others feel that more proof is necessary. It is possible that the slit-lamp biomicroscopy findings are sufficient for everyday clinical practice but that more evidence should be considered in the context of clinical trials. The presence of conjunctival or mixed epithelial phenotypes in the central cornea is proof of conjunctivalization or, in other words, LSCD (Figure 2 and Figure 4). Undoubtedly, demonstration of the corneal epithelial phenotype in at least the central cornea following treatment by a stem cell-dependent method can be considered as objective proof of restoration and validation of the LSCD diagnosis. Whether to prove the presence of LSCD by in vivo confocal microscopy or impression cytology is debatable, and each clinical center uses their available resources. However, it is also clear that the epithelium that develops after the transplantation should not be jeopardized by removing the 2–3 layers that corneal impression cytology requires. Anterior segment optical coherence tomography (OCT) is also a good tool to provide high resolution images of the limbal niche [111] and corneal abnormalities pre-, intra- and post-operatively [112,113]. OCT can be especially helpful in showing more clearly how deep the opacification of the cornea is in cases where a clinical inspection at the slit-lamp is unreliable. This allows the clinician to make a therapy plan and to apprise patients and referring physicians whether a further corneal transplantation is probably needed for visual recovery purposes after cell transplantation. The presence of corneal opacification deeper than the anterior stroma due to the etiology of the LSCD, e.g., chemical burn, is an obvious reason for which visual acuity alone is not an adequate parameter to judge the potential efficacy of stem cell-based techniques [37].

Among the many clinical considerations with these challenging patients is the importance of maximizing medical therapy before planning any surgical approaches. The ocular surface must be as quiet as possible for the delicate transplanted stem cells to survive and have a chance to be effective. Thus pre-, peri-, and post-transplant medications need to effectively reduce or eliminate, if possible, ocular surface inflammation. Thus, any eyedrop applied to the eye must be gentle, i.e., unpreserved, and, ideally, applied with a clearly planned protocol that is tailored to each specific case [34]. The issue of systemic immunosuppression for 6–12 months when transplanting allogeneic cells has been addressed above, although this is not a concern for allogeneic MSCs.

A particular aspect of medical treatment and how to best prepare the ocular surface for a future cell transplant is whether corneal neovascularization can be diminished before cell transplant. A related issue is when and how to deal with the remaining neovessels after transplantation. The currently used protocols for treating neovessels are rarely successful [114] and could affect stem cell viability; thus, there is uncertainty about the most adequate timing of treatments. However, the encouraging protocol developed by Yin and Jacobs using the Prosthetic Replacement of Ocular Surface Ecosystem (PROSE) as a delivery system for topical bevacizumab has shown spectacular long-term results [115], and it may be applied to LSCD cases before cell transplantation or even after.

An important strategic consideration prior to stem cell transplantation is to avoid concomitant surgeries that cause additional inflammation and place the transplanted stem cells at risk. For example, the repair of adnexal abnormalities associated with the health and function of the eyelids, conjunctival fornices, symblephara, etc., must be assessed and improved prior to cell transplantation to ensure the best chance of stem cell survival and epithelial healing. Cataract surgery, if needed, must be performed before if possible. Corneal transplantation must not be performed at the same time as stem cell transplantation, and all ocular procedures must wait until cell transplantation is considered finished, between 6 and 12 months [24].

Although the most common practice is to excise any conjunctival tissue encroaching upon the cornea just prior to placing the stem cells on the ocular surface, there is a debate about the best technique to prepare the recipient bed. Another concern is the level of aggressiveness that the ocular surgeon should take in certain etiologies, such as in Stevens–Johnson’s syndrome and its spectrum, where the response to surgical aggression can be enormous.

An important consideration, though rarely mentioned, is how to treat limbal scar tissue. Surgically removing it could stimulate neovessel encroachment upon the cornea; therefore, uncertainty exists regarding the best option, i.e., leave it in place or totally or partially removing it.

Clinicians also do not know with certainty if it is better to place the stem cell-based products with cells facing down, i.e., in close contact with tissues, or with cells facing up, i.e., with the carrier in contact with the tissues. There is no agreement or data supporting one way over the other, and each medical team fervently defends its position.

At this stage of clinical development, there are many questions that remain to be asked and answered. For instance, what is the best way to protect the transplant during the immediate post-operative period? Several approaches have been tried, e.g., scleral contact lens, partial tarsorrhaphy, and botulin toxin injection to promote ptosis. However, no protocols have been systematically designed and investigated.

In summary, better-designed randomized and parallel-controlled clinical trials at multiple centers are needed to address the clinical challenges presented here and others as well. Extensive follow-up is necessary to ascertain which technique is best for each specific clinical scenario, how long each type of transplant lasts, and how often each can be repeated. In addition, to help clinicians arrive at a consensus for each of the challenges described here, they need answers from scientists focused on the cellular and molecular aspects of treatments that show promising clinical results.

### 4.2. Preclinical Challenges

Despite the large number of clinical studies that have shown quite high success rates in different stem cell-based LSCD therapies, there are still many preclinical challenges to overcome and many questions to answer that could further improve the current and yet to come available techniques.

**Figure 6:**
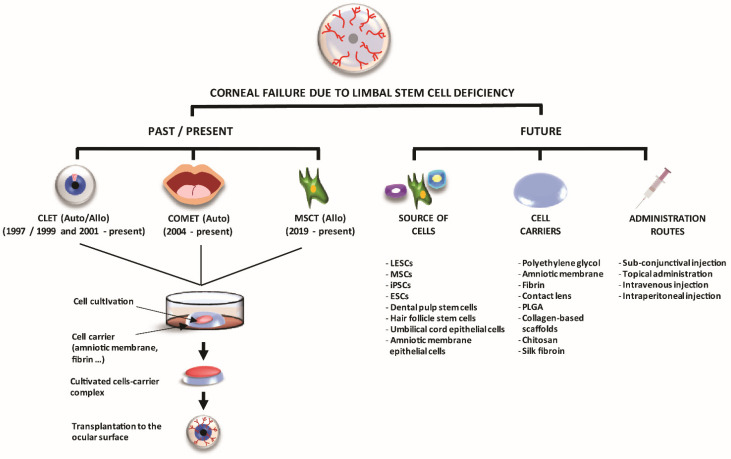
Representation of the different stem cell-based therapy techniques, sources of cells, cell carriers, and alternative administration routes that were used in the past, are being used at present, or will likely be used in the future. Auto: autologous; Allo: allogeneic; CLET: cultivated limbal epithelial transplantation; COMET: cultivated oral mucosa epithelial cells; ESCs: embryonic stem cells; iPSCs: induced pluripotent stem cells; LESCs: limbal epithelial stem cells; LSCD: limbal stem cell deficiency; MSCs: mesenchymal stem cells; MSCT: mesenchymal stem cell transplantation; PLGA: poly lactide-co-glycolic acid.

Among the remaining challenges, there is the fact that we still do not fully understand the mechanism by which the transplanted stem cells help repair the damaged ocular surface. Additionally, it is clear that transplantation of just a sheet of stem cells is not enough for reconstructing a damaged limbal stem cell niche. Therefore, alternative routes of cell administration and tissue-engineering techniques must be developed and investigated. Additionally, there is an ongoing research effort to find alternative sources of non-limbal stem cells that have not achieved clinical application but which have promising preclinical results with different degree of success that are also summarized in this section (Figure 6).

#### 4.2.1. Analysis of the Corneal Epithelium after Stem Cell-Based Transplantation

At present, the mechanism of action and the fate of the administered stem-cells are still uncertain. The possible cell fates include (1) being metabolized and degraded after application, (2) remaining embedded in the administration place, (3) migration to damaged tissues, or (4) other unknown fates. The mechanism(s) by which transplanted stem cells act could include replicating as stem cells, hopefully after they settle in their niche, and/or by delivering soluble factors into their milieu [34,116].

Although many of these questions are difficult to solve in humans, different techniques have been used in patients to identify the phenotype of cells at the ocular surface after a stem cell transplantation. For instance, the histologic characteristics of the corneal epithelium can be analyzed after the host corneal tissue has been replaced by a corneal transplantation performed in the months following stem cell transplantation [22].

For example, Sangwan et al. showed a normal stratified corneal epithelium in 15 corneal buttons from penetrating keratoplasties where limbal and conjunctival epithelial cells had been grafted in 125 patients with LSCD [117].

Corneal impression cytology has also been used to analyze the phenotype of ocular surface epithelium after a stem cell transplantation. In these specimens, the presence of corneal epithelial markers keratin K3 or K12, conjunctival epithelial goblet cells, and conjunctival epithelial markers K19 and MuC5AC can be identified to determine if the transplanted stem cells achieved a corneal, conjunctival, or mixed phenotype [33,91,118,119].

Currently, corneal in vivo confocal microscopy has mostly replaced corneal impression cytology to study the quality of the new epithelium after stem cell transplantation. This technology is currently used not only to recognize the epithelium phenotype in the central corneal but also to analyze the limbal niche (Figure 2 and Figure 4). When performed on the cornea, the basal epithelium phenotype can be defined as corneal, conjunctival, or mixed. Additionally, inflammatory cells, such as dendritic cells and leucocytes, can be imaged as well as some other structures such as corneal nerves, limbal palisades of Vogt, blood vessels, etc. [33,120,121,122]. Several authors showed that the confocal microscopy results are correlated with the data obtained by impression cytology [91] with a concordance of 77% (10/13 eyes) [33]. However, the histological study of the ocular tissues obtained from a clinically necessary penetrating keratoplasty should not be replaced by in vivo confocal microscopy, as both techniques are complementary and both have been performed in parallel in recent years [22,24,33,47,89,94,117,122,123].

It is important to know the correlation between the characteristics of stem cell-based grafts and the success of the treatment. Rama et al. showed that 78% of the eyes with stem cell transplants were successful when more than 3% of the transplanted cells were positive for the limbal epithelial stem cell marker p63 [36]. The estimate of p63-positive cells was based on cells in primary culture, but the transplanted cells were harvested from secondary cultures that were derived from the primary cultures [36]. Later, the same authors confirmed that the number of clonogenicity cells, colony size, growth rate, and presence of conjunctival cells in grafts is not directly correlated with clinical outcomes [124].

Several groups have also analyzed the cell phenotype of cultured stem cells before developing a clinical transplantation protocol with the harvested cells [42,93,94,97,117,119,125]. For example, Zakaria et al. reported the expression of ABCG2, ΔNp63, and K14 markers in more than 50% of the amniotic membrane–limbal epithelial cell grafts [94].

Other researchers have used “replicated grafts” to analyze the expression of different markers [89,98]. Replicated grafts are cultures obtained in parallel to the graft to be transplanted; thus, they are composed of cells taken at the same culture passage and grown under the same GMP conditions as the actual graft cells. Replicated grafts, as surrogates for grafts that were actually used in clinical transplants, were analyzed for cell markers in cultured allogeneic limbal cells and allogeneic MSCs cultured on amniotic membrane [89]. The authors showed that 80% of the cells in both cell type cultures expressed the limbal cell markers K15 and p63alpha and corneal marker K3.

In summary, although there are many studies in which the markers and quality of the transplanted stem cells have been studied, there are inherent limitations to the investigation of the mechanism(s) of action and fate(s) of transplanted stem cell-based medicines in humans. As moral and ethical considerations restrict the in vivo use of humans as research models, other approaches must be developed to ask and answer the important questions about the use of stem cells for treating human diseases. Preclinical research tries to answer all of these questions to help clinicians offer patients better cell therapy products.

#### 4.2.2. The Need to Reconstruct the Limbal Stem Cell Niche

The well-known importance of the limbal niche in stem cell regulation, maintenance, proliferation, and differentiation has prompted researchers to seek the best substrata for the culture and transplantation of stem cells, trying to mimic the limbal natural niche. Several types of materials, both natural, such as human amniotic membrane or fibrin, and synthetic, such as poly lactide-co-glycolic acid (PLGA) or siloxane, have been developed over the years to facilitate the cell culture, handling, and corneal regeneration as well as to protect the graft itself (Figure 6).

Currently, the most frequently used substratefor stem cell culture and transplantation of cultured cells into patients with LSCD is human amniotic membrane. This tissue has unique characteristics, including anti-angiogenic, anti-inflammatory, and anti-bacterial properties, which help in the corneal regeneration process [126,127]. Moreover, the culture of limbal epithelial stem cells on amniotic membrane promotes the expression of stem cell markers such as p63α and ABCG2 and reduces some corneal differentiated markers such as K3, K12, and Cx43 [128]. Thus, the amniotic membrane has been successfully used in multitude of clinical studies [24,28,45,46,89,91,93,128,129,130,131,132,133,134,135].

Another natural carrier used in corneal regeneration is fibrin, previously isolated from human plasma [136]. This material has been widely used in ophthalmology as a surgical adhesive and as a biodegradable carrier for tissue engineering [137,138,139]. In fact, the first CLET was performed using fibrin as a carrier [22], and subsequently, many other studies have used it as a scaffold [36,124,140,141]. Additionally, the EMA-approved product Holoclar also uses fibrin in its composition [37,38].

Apart from natural products, some synthetic materials have also been developed for clinical use since 2007. One of these is a contact lens, Lotrafilcon A siloxane hydrogels, which serves as a cell carrier that supports limbal cell culture and expansion [48,142]. Contact lenses have some benefits compared with natural carriers, as they are transparent and easy to use in ophthalmology, the production is standardized, and some of these materials are commercially approved. However, these substrata are not biodegradable, and the use of contact lenses has not become widely accepted.

Additionally, synthetic biodegradable PLA polymers have been recently used for the treatment of LSCD by serving as a substratum for freshly excised limbal tissue [143]. The use of PLGA membranes as substitute for amniotic membranes or fibrin for limbal epithelial transplantation is a novel technique that will potentially benefit patients, reduce costs, and avoid the risk of disease transmission. A clinical trial completed by Sangwan et al. in 2018 (ClinicalTrials.gov: NCT02568527), evaluated the safety and efficacy of PLGA scaffolds to regenerate limbal epithelial stem cells for autologous limbal grafts in five patients with total unilateral LSCD. No results concerning this study have yet been published.

Several preclinical studies have been developed by using different biomaterials as carrier substrata for limbal cells. Among them are collagen-based scaffolds composed of type I or type IV collagen. They are nontoxic and support corneal epithelial cell culture in animal models. However, they have not yet been implemented in vivo in humans [144,145,146,147]. A variation of these materials is plastic compressed collagen, which has been used for co-cultures of limbal epithelial cells and fibroblasts [148,149,150]. This substratum simulates an artificial stroma and increases the capacity of limbal epithelial cell expansion.

Chitosan, a polysaccharide obtained from natural chitin, is a biocompatible, non-toxic, and bioresorbable polymer with antibacterial properties. It has been widely used as a carrier for limbal epithelial cells in preclinical studies [151,152,153]. De la Mata et al. demonstrated that glutaraldehyde-crosslinked chitosan, functionalized with gelatin, was suitable for the expansion and maintenance of human stem cells derived from the limbal niche, cultivated with non-xenogeneic supplements [151].

Silk fibroin has also demonstrated the potential to support the culture of both human and rabbit limbal epithelial cells [154]. Moreover, silk fibroin modified with polyethylene glycol was recently established as a potential carrier for limbal cell transplantation in rabbits [155]. Other synthetic polymers such as polyethylene glycol and some temperature-responsive substratum, such as poly(*N*-isopropylacrylamide), which facilitates cell adhesion, spreading, and growth, have been successfully used in preclinical studies to analyze their potential as carriers for further cell transplantation [156].

All of these potential carriers can be considered opportunities to improve the currently available LSCD therapies. The use of a substratum serves not only to facilitate the process of cell transplantation but also to simulate the limbal niche, providing cells with an ideal environment to grow, proliferate, and maintain their phenotype. Additionally, combining different sources of cells, such as MSCs and others, together with appropriate biological scaffolds, appears to be a promising strategy for long-term revitalization of the limbal niche [157].

In summary, although many different substrata have been investigated with the initial idea of providing an environment similar to the limbal niche, a realistic approach has not yet been achieved in the reconstruction of the architecture of the human healthy limbal niche.

#### 4.2.3. Alternative Routes of Delivering Stem Cell-Therapy

The most commonly used route of stem cell administration to treat LSCD is by affixing with sutures or biological glues the cultivated cells onto the top of the damaged cornea and limbal areas. However, other routes of administration have been studied, some of them with promising results (Figure 6). Topical administration of cells is one of the easiest ways to apply them to the ocular surface. It avoids the need of carriers, surgical sutures, or glues. Indeed, it avoids the whole surgical procedure. However, there are some drawbacks to topical application of the stem cells, such as low retention time, high washing off rate, and low penetration of the corneal epithelium.

The topical administration of MSCs provides therapeutic and anti-inflammatory effects in different experimental models of corneal epithelial damage [79,158]. A clinical trial performed by Boto et al. (ClinicalTrials.gov: NCT01808378) administered adipose tissue-derived MSCs (AT-MSCs) topically in combination with subconjunctival injection of AT-MSCs; however, the results of this study have not yet been published. A clinical trial by Auffarth et al. (ClinicalTrials.gov: NCT03549299), active since 2018 but not yet recruiting, proposes topical application of four different doses of allogeneic ABCB5-positive limbal stem cells. The results from these and future clinical trials are needed to determine if topical administration of cells can be a fully effective route of administration for ocular surface treatment.

The subconjunctival injection of cells is another local route of administration that has been extensively studied. This technique has numerous advantages: (1) It is minimally invasive and does not require a surgical facility. (2) The cell product can be prepared easily, and carriers are not needed. (3) It allows for the administration of high cell doses in a small volume. (4) The cell dose administered can be effectively controlled. (5) It can be used in severe cases of LSCD [159]. However, a large volume of solution cannot be injected into the subconjunctiva, and there are yet no consensus on the best vehicle solution, the number and location of injections, and the dose of cells to be administered. In spite of this, there are several works showing that the subconjunctival injection of MSCs reduces inflammation of the ocular surface in different experimental models of corneal epithelial damage [80,160,161]. It also reduces clinical signs such as corneal neovascularization, opacity, and epithelial defects [162,163,164]. The most frequently injected cell type is MSCs, but oral mucosal epithelial cells have also been subconjunctivally injected in a rat experimental LSCD model [165].

After the transplantation of expanded cells on a human amniotic membrane or fibrin, the subconjunctival injection of AT-MSCs or bone marrow-derived MSCs (BM-MSCs) for the treatment of corneal epithelial damage is the most studied technique in humans. To date, three clinical trials have been completed (ClinicalTrials.gov: NCT01808378, NCT04484402, and NCT02325843) but not yet published. Additionally, there are two other clinical trials registered in which umbilical cord MSCs or allogeneic BM-MSCs will be injected subconjunctivally (ClinicalTrials.gov: NCT03237442 and NCT03967275). Although more clinical evidence is needed to determine if subconjunctival injection is an effective route of cell administration for the treatment of LSCD, the advantages and preclinical evidence makes it one of the most promising techniques.

Another option in preclinical study phases for the treatment of ocular surface pathology is the systemic injection of stem cells. The ability of MSCs to migrate into damaged or inflamed tissues [166] makes MSCs the most suitable cell type for systemic administration in the treatment of LSCD. However, systemic administration presents a high risk of side effects, and the number of cells that reach the target tissue is low [65,167]. Moreover, as with all cell administration by injection, there is still no consensus regarding the best vehicle solution. However, in contrast with subconjunctival injection, systemic administration allows for large volumes to be administered and much higher cell doses can be achieved [159].

Both intraperitoneal and intravenous administration of BM-MSCs have reduced corneal opacity [64,65,74,168,169] and ocular surface inflammation [65,75,169] in different experimental models of corneal epithelial damage. However, to the best of our knowledge, these routes of administration have not been used in humans yet for the treatment of corneal failure due to LSCD.

In summary, interesting new alternative routes and doses could undergo further preclinical investigations before finally being translated into clinical use.

#### 4.2.4. Other Potential Sources of Non-Limbal Cells

As described above, limbal epithelial cells have limitations with respect to providing a sufficient number of stem cells to ensure successful treatment of LSCD. For that reason, other cell sources have been sought. Up to now, oral mucosa epithelial cells and MSCs are the only non-limbal epithelial cells that have been proven to be safe and effective for treating patients with ocular surface failure due LSCD [55,89]. Nevertheless, during the last few years, other alternative sources of non-limbal cells have also been investigated in experimental studies with different degrees of success [34,170,171,172] (Figure 6).

##### Embryonic Stem Cells

Human embryonic stem cells (hESCs) are pluripotent stem cells derived from the inner cell mass of human blastocysts, and they can differentiate into derivatives of all three germ layers [173]. Therefore, the differentiation of hESCs into corneal or limbal epithelial cells offers the potential of an unlimited source of cells to treat patients suffering from LSCD.

The significance of reproducing the corneal stem cell environment to induce hESCs to differentiate towards a corneal or limbal epithelial-like cell phenotype has been supported by several studies [174,175,176,177]. By mimicking the microenvironment of the corneal epithelial stem cell niche in vitro, Ahmad et al. successfully induced hESC differentiation into corneal epithelial-like cells. To that end, they cultured hESCs on type IV collagen, a component of the corneal epithelial basement membrane, using media conditioned by limbal fibroblasts [177]. Other authors differentiated hESCs into corneal epithelial-like cells by seeding them onto a partially de-epithelialized or de-cellularized human corneal buttons [174]. Others coaxed the hESCs to develop into limbal epithelial stem cell-like cells by using a culture medium conditioned by limbal epithelial stem cells [175]. However, all of these differentiation techniques depend on corneal tissue donors to either culture the hESCs on them or to prepare the conditioned media. With the potential biological variations among tissues from different donors, this greatly limits the potential of these differentiation techniques.

To overcome these limitations, Zhang et al. developed a protocol to differentiate hESCs into corneal epithelial progenitor cells using a defined serum-free medium [178]. They demonstrated the functionality of the progenitor cells grown on an acellular porcine corneal matrix and transplanted onto rabbit eyes [179]. Very recently, He et al. have also demonstrated that clinical-grade hESC-derived corneal epithelial cell sheets successfully helped repair the damaged ocular surface of a rabbit LSCD model [180].

Despite being a potential limitless source of cells, the future clinical use of hESCs for treating patients with LSCD might be hampered due to some drawbacks, such as the ethical controversy regarding their embryonic origin, their immunogenicity, and their potential tumorigenicity [181].

##### Induced Pluripotent Stem Cells

Human-induced pluripotent stem cells (hiPSCs), which have characteristics that are similar to hESCs, are generated by manipulation of differentiated adult cells [182]. Since the reprogramming technique was described, great efforts have been made to generate corneal and limbal epithelial cells from hiPSCs. This technology could provide an unlimited supply of limbal and corneal epithelial cells without any ethical issue for treating patients with LSCD (reviewed in [183,184,185]).

Hayashi et al. reported the first method to generate corneal epithelial cells from hiPSCs derived from both adult corneal limbal epithelial cells and human dermal fibroblasts [186]. Later, these same authors described a strategy to generate corneal stem and progenitor cells from hiPSCs by reproducing in vitro the whole eye development. Using this approach, they generated an epithelial cell sheet that successfully restored corneal function in a rabbit model of LSCD [187,188]. Using small molecules, Mikhailova et al. developed a directed two-stage differentiation protocol to generate corneal epithelial-like progenitor cells with the capacity to terminally differentiate towards mature corneal epithelial-like cells [189]. This same research group later published another reproducible and clinical compatible differentiation method using xeno-free conditions to generate limbal epithelial stem cells from hiPSCs [190]. Apart from the ones already mentioned, several other methods have been published with the aim to generate functional corneal and limbal epithelial cells from hiPSCs (reviewed in [183,184,185]). However, before translating these methodologies to clinical applications, further improvements must be made on the derivation protocols because they are extremely expensive. They also require a considerable amount of time, first, for hiPSC generation and, later, for corneal and limbal cell induction. The creation of HLA-typed hiPSC banks has been proposed as a potential solution to partially overcome these two limitations and to also reduce the potential problems of immunogenicity [191,192]. Furthermore, given that hiPSC generation could induce mutagenicity, extensive genetic analyses must be performed on the hiPSCs intended for transplantation into patients to ensure genetic fidelity and stability [193]. Among these, reprogramming vector analysis and karyotype analysis are considered mandatory, while performing single nucleotide polymorphism arrays, whole genome analysis, and other genetic and disease marker analyses are considered to be for informational purposes only [193]. Considering that the tumorigenic potential of hiPSC-derived corneal epithelial cells has not been fully tested [194], the likelihood of it cannot be conclusively ruled out due to the possible presence of undifferentiated or partially differentiated iPSCs in the cell population intended to be transplanted [194]. Consequently, the development of protocols to directly reprogram adult cells towards a designated phenotype, thus avoiding the pluripotent state, could help to diminish the tumorigenicity of the transplanted cells [195]. In this context, a direct transdifferentiation protocol to generate corneal/limbal epithelial cells from human dermal fibroblasts has already been published [196]. Therefore, this new methodology that bypasses the hiPSC stage might provide a safer source of corneal epithelial cells devoid of tumorigenic potential.

##### Dental Pulp Stem Cells

Human immature dental pulp stem cells isolated from exfoliated teeth express both MSC, ESC, and limbal epithelial stem cell markers such as p63 and ABCG2. The transplantation of these stem cells into a LSCD rabbit model reduced corneal neovascularization and conjunctivalization, and reconstructed the damaged ocular surface by developing a well-formed corneal epithelium that expressed limbal epithelial stem cell and corneal epithelial cell markers [197,198]. These and other authors further developed the capacity of dental pulp stem cells to acquire corneal and limbal epithelial features using different cell culture techniques [199,200] and cell carriers [201,202]. However, although these cells seem to represent a valid alternative source of cells for treating patients with LSCD, more preclinical evidence should be gathered, especially related to their tumorigenicity, before translating this technology into clinical practice.

##### Hair Follicle Bulge-Derived Epithelial Stem Cells

Hair follicle bulge-derived stem cells are a population of epithelial stem cells involved in forming hair follicles and in regenerating the epidermis during wound healing [203,204]. Blazejewska et al. demonstrated that these cells were also able to differentiate into corneal epithelial-like cells. They reported that, by mimicking the limbal microenvironment, hair follicle bulge-derived stem cells cultivated on laminin-5 in a culture medium conditioned by limbal fibroblast showed the structural, morphological, and molecular features of corneal epithelial cells [205]. Later, the functionality and therapeutic potential of these stem cells was further confirmed in a mouse model of LSCD [206]. Mainly because of their easy accessibility, hair follicle bulge-derived epithelial stem cells might be an interesting alternative source of stem cells for treating LSCD. However, apart from these initial studies, not much further work has been performed.

##### Amniotic Membrane Epithelial Cells

The inner surface of the human amniotic membrane is covered by a continuous single layer of ectodermal cells, the amniotic membrane epithelial cells. These cells not only express hESC markers and pluripotent stem cell-like characteristics but also have a MSC-like phenotype. As a consequence, they also have both low immunogenicity and high immunomodulatory properties [207,208]. Studies have reported the potential of human amniotic membrane epithelial cells to differentiate into corneal epithelial cells and to reconstruct the damaged ocular surface of rabbit LSCD models [209,210,211,212]. Therefore, this type of cell represents another alternative source of stem cells for treating LSCD, and similar to other potential sources of stem cells other than hESCs, their use does not provoke any ethical issues. It is important to highlight that these cells are also genetically stable because they do not form tumors upon transplantation into immunodeficient mice [207]. However, the data regarding the mechanism by which these cells exert their immunomodulatory properties is still not fully known. That is why further studies to elucidate the mechanism underlying this effect are required before they are used for clinical purposes [208].

##### Umbilical Cord Lining Epithelial Cells

Human umbilical cord lining epithelial cells are a population of pluripotent stem cells that express a cytokeratin pattern similar to human epidermal cells [213] and are capable of forming stratified epithelial sheets [214,215]. Reza et al. investigated the effectiveness of a mucin-expressing cell line derived from the cord lining for treating LSCD in a rabbit model using denuded human amniotic membrane as a cell carrier [216]. The mucin-expressing cord lining epithelial cells regenerated the damaged ocular surface with minimal neovascularization and opacification and formed a stratified epithelium that expressed the corneal epithelial markers K3/12. These cells showed no tumorigenicity, and there was no immune rejection, indicating low immunogenicity. Nevertheless, additional studies must be performed to verify those results before using this source of cells for treating patients.

Besides the cell-based therapies that are currently used in clinical practice, an increasing range of alternative sources of stem cells is being investigated. Many highly varied inducing protocols have been published to culture and later differentiate all of these types of human stem cells into corneal and limbal epithelial cells. However, there are no studies that compare the outcomes obtained among the different methodologies, and as a consequence, there are no standardized differentiation protocols. Therefore, efforts should be made to elaborate standard and reproducible differentiation protocols for each of the different types of stem cells [185]. There is also a need to develop cell culture techniques with chemically defined xeno-free culture media that meet the clinical grade requirements. This will increase the reproducibility, the quality, and the safety of the final stem cell-based product. Although any of these alternative stem cell sources could become a successful therapy for treating ocular surface failure due to LSCD, there are still some other challenges, such as tumorigenicity and immunogenicity that need to be overcome.

### 4.3. Regulatory Challenges

Stem cell-based therapies for corneal blindness due to ocular surface failure must meet the same regulatory requirements as other cell-based products for any other indication. Under most of the regulatory frameworks, this means that, among other requirements, the production of the cell-based product must comply with the principles of GMP. Furthermore, human clinical trials must be designed and conducted in compliance with the principles of good clinical practice. As the regulatory procedure to obtain approval of a cell-based therapy requires a lot of expertise, time, and investment, the process of bringing a new stem cell-based product to the clinic is very challenging. This is especially significant for small developers, such as universities, hospitals, and small- to medium-sized enterprises for which compliance with the regulations of cell-based products can be difficult and unaffordable. To this end, several agencies from different jurisdictions, such as the ones from the EU, Japan, and the US, have implemented mechanisms to accelerate and optimize the development of this type of medicinal product. The compliance with national and international regulations is vital for the development and commercialization of these therapies. Therefore, one of the greatest regulatory challenges for the coming years will be to establish an international harmonization of the regulatory frameworks that control the development of stem cell-based medicinal products throughout the globe [38].

## 5. Conclusions

The application of surgical therapies to treat corneal pathology due stem cell destruction, just a decade after the discovery of stem cells at the ocular surface, has made an extraordinary progress over the last three decades. A new name, LSCD, was given to the corneal failure caused by a wide variety of pathologies that can destroy the corneoscleral limbus. Transplantation of limbal tissues (either whole or minced limbal segments) began and was followed some years later by stem cell-based transplants in an effort to repair these severe clinical entities.

At present, to avoid the need for immunosuppression, only unilateral cases of LSCD are eligible for autologous transplantations, either tissue-based or cultivated stem cell-derived. Bilateral severe cases can benefit from autologous transplants only when extraocular stem cells can be used. For that purpose, only COMET has been translated into clinical practice. Another alternative in bilateral LSCD cases is to use allografts. In the case of whole or minced limbal tissue tissue-based allografts, long-term immunosuppression is needed to avoid immune rejection. Short-term immunosuppression is needed if cultivated stem cell-based therapies, such as allogeneic CLET, are planned. However, to avoid host immunosuppression, immune-privileged stem cells such as allogeneic MSCs, the most often used stem cell type in regenerative medicine, has just entered the ophthalmic armamentarium. As only allogeneic therapies are of interest for commercialization purposes at affordable prices, more efforts must be made by both research and clinical scientists to develop medicinal products based on allogeneic sources. Even with all of the progress made thus far, we must be aware that, while current therapies are delivering stem cells or stem cell-rich products onto the cornea and limbal area, the limbal niche itself has not yet been reconstructed.

In summary, only the close collaboration between preclinical and clinical scientists, international regulatory agencies, governmental and non-governmental financial sources, and the pharmaceutical industry make meaningful achievements that can effectively reach patients affected by LSCD pathologies, making the statement “from the bench to the bedside” truer than ever.

## Figures and Tables

**Table 1:** Published studies using stem cell-based transplants compared with other techniques (in at least five eyes) in the management of severe limbal stem cell deficiency (LSCD).

Publication’s First Author, Year (Reference No.) Country	Type of Clinical Study/No. Surgeries or Eyes/Mean Time to Final Evaluation (months)	Type of Transplant (n), GMP ^1^ Followed for Product Preparation	Systemic Immunosuppressants in Allogeneic Transplants	Anatomical Success (Method of Evaluation)/Statistically Significant (s), Non-Significant (ns) or Not Mentioned (nm)
Shimazaki et al., 2007 [90] Japan	Retrospective, observational case series/27/31.6	Auto ^2^-CLET ^3^ (7) Allo ^5^-CLET (20) No	CsA ^4^ for 6 months	Global: 59.3% (clinical) Auto-CLET 85.7% Allo-CLET: 50.0%/ns
Shortt et al., 2008 [91] UK	Prospective, noncomparative, interventional case series/10/6 or 13	Auto-CLET (3) Allo-CLET (7) Yes	CsA for 6 months	Global: 60% (clinical, ccp-IVCM ^6^, impression cytology) Auto-CLET: 33% Allo-CLET: 71%/nm ^7^
Pauklin et al., 2010 [92] Germany	Prospective noncomparative interventional case series/44/28.5	Auto-CLET (30) Allo-CLET (14) No	CsA for 12–15 months (one case had none)	Global: 68% (clinical) Auto-CLET: 76.7% Allo-CLET: 50%/s
Prabhasawat et al., 2012 [93] Thailand	Prospective, noncomparative case series/19/26.1	Auto-CLET (12) Allo-CLET (7) No	CsA for 6–12 months	Global: 73.7% (clinical) Auto-CLET: 66.7% Allo-CLET: 85.7%/ns
Zakaria et al., 2014 [94] Belgium	Phase I-II non-randomized clinical trial/18/22	Auto-CLET (15) Allo-CLET (3) No	CsA for 12 months	Global: 66.7% (clinical) Auto-CLET: 66.7% Allo-CLET: 66.7%/ns
Ramírez et al., 2015 [24] Spain	Prospective noncomparative interventional case series/20/12, 24, 36	Auto-CLET (11) Allo-CLET (9) Yes	Mycophenolate CsA or azathioprine for 12 months	Global (clinical, ccp-IVCM): 80% at 1–2 years; 75% at 3 years Auto-CLET: 90.9% Allo-CLET: 66.7%/ns
Ganger et al., 2015 [95] (India)	Retrospective case series/62 (38 children, 24 adults)/12	Auto-CLET (54) Allo-CLET (8) No	No	Global: nm Auto-CLET: 87.8% children, 99.9% adults/ns Allo-CLET: 62.5%/ns
Parihar et al., 2017 [96] India	Prospective interventional/50/12	Allo-CLET (25) Allo-LTT ^8^ (25) No	CsA for 12 months	Global (clinical): nm Both groups had significant improvement/ns
Sharma et al., 2018 [97] India, USA, Australia	Prospective comparative/40/12	Auto-CLET (20) AMT ^9^ (20) No	Na ^10^	Global (clinical): nm Similar results in both groups
Calonge et al., 2019 [89] Spain	Phase I-II, randomized, controlled, double-masked clinical trial/28/12	Allo-MSCT ^11^ (17) Allo-CLET (11) Yes	Yes	Global (clinical ccp-IVCM): Allo-MSCT: 85.7% Allo-CLET: 77.8%/ns MSCT was as safe as CLET
Campbell et al., 2019 [98] UK	Randomized, controlled, single-masked, multicenter clinical trial/16	Allo-CLE (11) AMT (5) Yes	Prednisolone plus CsA or mycophenolate for 12 months	Global (clinical): nm sustained significant improvement in allo-CLET but not in AMT
Borderie et al., 2019 [47] France	Phase II, noncomparative clinical trial: CLET vs. retrospective control: LTT/30 /72 vs. 132	Auto-CLET (7) Allo-CLET (7) Auto-LTT (8) Allo-LTT (8) No	Allo-CLET: NO Allo-LTT: CsA, steroids or chloraminophen for 12 months	Global survival (clinical) at 5 years/nm Auto-CLET: 71% Allo-CLET: 0% Auto-LTT: 75% Allo-LTT: 33%
Wang et al., 2019 [58] China	Retrospective cohort study/76/23.3 vs. 16.1	Allo-CLET (42) Auto-COMET ^12^ (34) No	No (only oral corticosteroids for 2–3 months)	Global (clinical): nm Allo-CLET: 71.4% (Immune rejections: 9.5%) COMET: 52.9%)
Behaegel et al., 2019 [99] Belgium	Prospective, noncomparative case series (first 2 years); Later follow-up or retrospective review/13/2.1 (short-term) vs. 6.7 (long-term)	Auto-CLET (9) Allo-CLET (4) Yes	Not specified	Global short-term: 46.1% Auto-CLET: 77.8% Allo-CLET: 75% Global long-term: 23.1%/ns Auto-CLET: 55% Allo-CLET: 0% Success decreased over time
Shimazaki et al. [100] 2020 Japan	Retrospective analysis/246/89.3	Auto-CLET + auto-COMET (171) Allo-CLET (75) No	CsA for unknown period	Global (clinical): 65.1% Auto-CLET + COMET: 65.6% Allo-CLET: 63.0%/ns

^1^ GMP, good manufacturing practice. ^2^ Auto, autologous. ^3^ CLET, cultivated limbal epithelial transplantation. ^4^ CsA, cyclosporin A. ^5^ Allo, allogeneic. ^6^ ccp-IVCM, central corneal phenotype by in vivo confocal microscopy. ^7^ nm, not mentioned. ^8^ LTT, limbal tissue transplantation. ^9^ AMT, amniotic membrane transplantation. ^10^ na, non-applicable. ^11^ MSCT, mesenchymal stem cell transplantation. ^12^ COMET, cultivated oral mucosal epithelial cell transplantation.

## Data Availability

Publicly available datasets were analyzed in this review. This data can be found here: https://www.clinicaltrials.gov/ (accessed on 12 July 2021).
